# Insights From fMRI Studies Into Ingroup Bias

**DOI:** 10.3389/fpsyg.2018.01868

**Published:** 2018-10-01

**Authors:** Pascal Molenberghs, Winnifred R. Louis

**Affiliations:** ^1^School of Psychological Sciences, The University of Melbourne, Melbourne, VIC, Australia; ^2^School of Psychology, The University of Queensland, Brisbane, QLD, Australia

**Keywords:** ingroup bias, intergroup violence, fMRI, social neuroscience, empathy, mentalizing, morality

## Abstract

Intergroup biases can manifest themselves between a wide variety of different groups such as people from different races, nations, ethnicities, political or religious beliefs, opposing sport teams or even arbitrary groups. In this review we provide a neuroscientific overview of functional Magnetic Resonance Imaging (fMRI) studies that have revealed how group dynamics impact on various cognitive and emotional systems at different levels of information processing. We first describe how people can perceive the faces, words and actions of ingroup and outgroup members in a biased way. Second, we focus on how activity in brain areas involved in empathizing with the pain of others, such as the dorsal anterior cingulate cortex (dACC) and anterior insula (AI), are influenced by group membership. Third, we describe how group membership influences activity in brain areas involved in mentalizing such as the medial prefrontal cortex (mPFC) and temporoparietal junction (TPJ). Fourth, we discuss the involvement of the lateral orbitofrontal cortex (lOFC) in increased moral sensitivity for outgroup threats. Finally, we discuss how brain areas involved in the reward system such as the striatum and medial orbitofrontal cortex (mOFC), are more active when experiencing schadenfreude for outgroup harm and when rewarding ingroup (versus outgroup) members. The value of these neuroscientific insights to better understand ingroup bias are discussed, as well as limitations and future research directions.

## Introduction

There is increasing neuroscientific evidence that people process information from ingroup and outgroup members in a different way (for reviews see: Ito and Bartholow, 2009; Cikara et al., 2011b; Kubota et al., 2012; Eres and Molenberghs, 2013; Molenberghs, 2013; Amodio, 2014; Cikara and Van Bavel, 2014; Han, 2018; Mattan et al., 2018). Biases toward outgroup members affect perceptions, attitudes and behaviors. As such, the whole brain can be involved in ingroup bias processes but specific patterns depend on which modality is involved (Molenberghs, 2013). To better understand and predict ingroup biases, and how they can lead to ingroup favoritism and intergroup violence, it is critical to build better multidisciplinary psychological moral models that are grounded in biological reality (Van Bavel et al., 2015). This requires a multidisciplinary integration of information from evolutionary theory, psychology, political science and neuroscience (Decety et al., 2017). The aim of this review is to provide an overview of some the most important insights from fMRI studies into biased processing of ingroup and outgroup members, to better understand ingroup bias.

**FIGURE 1:**
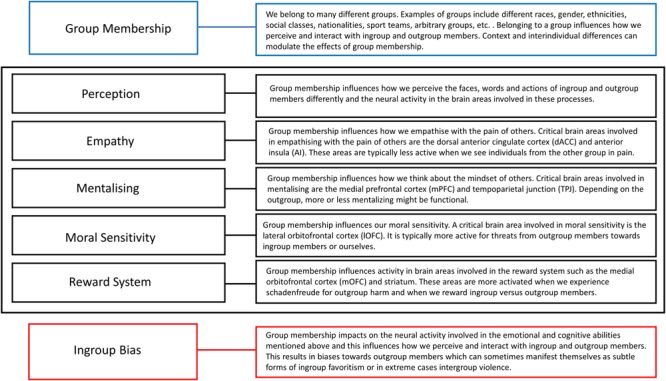
A schematic overview of how group membership can influence activity in the brain areas involved in perception, empathy, mentalizing, moral sensitivity and the reward system as discussed in this paper. Together, these modulations in neural activity can lead to ingroup bias.

We first describe how group membership influences neural activity involved in face, word and action perception, and how this leads to perceiving ingroup and outgroup members in a biased way. Then we focus on empathizing with the pain of others and our reduced neural sensitivity for watching outgroup members in pain. The third section describes how group dynamics influence activity in brain areas involved in our ability to think about the mindset of others. The next section discusses the role of the lOFC in our heightened sensitivity for outgroup threats. Finally, we describe how brain areas involved in our reward system show increased activation when feeling schadenfreude in response to outgroup harm or when rewarding ingroup versus outgroup members. There are several other ways in which group dynamics can influence the neural mechanisms subserving our emotional and cognitive abilities involved in ingroup bias, but the ones described above have been studied enough in detail with multiple fMRI studies and will therefore be the focus of this review. **Figure 1** provides a schematic overview of the five ways how group membership can influence information processing in the cognitive functions and neural systems mentioned above, and which are discussed in detail in the following sections. Together they influence how people perceive ingroup and outgroup members and act toward them. These effects are influenced by context and individual differences and can sometimes manifest themselves as subtle forms of ingroup favoritism, or in extreme cases, intergroup violence.

## Biased Perception of Faces, Words and Actions

Functional magnetic resonance imaging studies have revealed different neural responses to perceiving faces of ingroup and outgroup members (e.g., Hart et al., 2000; Phelps et al., 2000; Golby et al., 2001; Richeson et al., 2003; Lieberman et al., 2005; Kaplan et al., 2007; Chiao et al., 2008; Van Bavel et al., 2008, 2011; Freeman et al., 2010; Liu et al., 2015), and these effects seem to be task dependent. For example, Cunningham et al. (2004) found that when Caucasian participants watched Black and White faces that were presented very briefly (i.e., 30 ms, so that they were barely a flash on the screen), they showed increased activation in the amygdala in response to the Black faces (which was interpreted as an increased emotional response for outgroup faces). However, this effect disappeared when the pictures were presented for a longer time so that they were clearly visible (i.e., 525 ms). In addition, the prefrontal cortex was more active for White faces compared to Black faces in this condition. The authors suggested that the longer stimulus presentation in this latter condition, allowed the participants to regulate the automatic implicit amygdala bias observed in the former condition through increased prefrontal cortex activation, because participants did not want to be biased, or perceived as biased (Cunningham et al., 2004).

Wheeler and Fiske (2005) also found that amygdala responses were influenced by different face perception tasks. Caucasian participants showed more activation in the amygdala during a social categorization task (i.e., a task where participants had to categorize people based on their age) when observing Black compared to White faces. However, no group difference was found in a non-social visual search task (i.e., a task in which people had to detect whether a dot was present on someone’s face), and more amygdala activation for White compared to Black faces was found during an individuation task (i.e., a task in which participants had to think about what the person liked). The authors concluded that: (1) the simple social categorization task resulted in the increased automatic emotional prejudice response for outgroup targets and thus increased amygdala activation; (2) the faces during the non-social visual search task were not processed deeply enough to represent a social target, and therefore no group effect was observed in the amygdala during this task; and (3) the individuation task resulted in deep level controlled processing and thus a suppression of amygdala responses to outgroup targets as seen in the Cunningham et al. (2004) study.

Another brain region modulated by group membership during face perception is the fusiform face area (FFA). Using fMRI, Golby et al. (2001) showed that this region responds more strongly to same-race faces compared to other-race faces. However, in another fMRI experiment it was shown that when White participants were randomly assigned to a novel mixed-race team, they showed more FFA activity for ingroup team (vs. outgroup team) faces regardless of their race (Van Bavel et al., 2008). In a similar follow-up experiment, which also included a mixed-race control condition with faces that did not belong to the ingroup or outgroup, Van Bavel et al. (2011) again showed enhanced FFA activity for ingroup (vs. outgroup) faces regardless of race (but see Ratner et al., 2012). By comparing the ingroup and outgroup conditions with the control condition, they showed that this increase was caused by enhanced FFA activity for the ingroup faces rather than diminished FFA activity for the outgroup faces. Together these fMRI studies on face perception show that people can regulate the increased amygdala response for outgroup faces (Cunningham et al., 2004; Wheeler and Fiske, 2005), and that we activate the FFA more for faces of ingroup members (Golby et al., 2001), regardless if they are the same race or not (Van Bavel et al., 2008, 2011).

**FIGURE 2:**
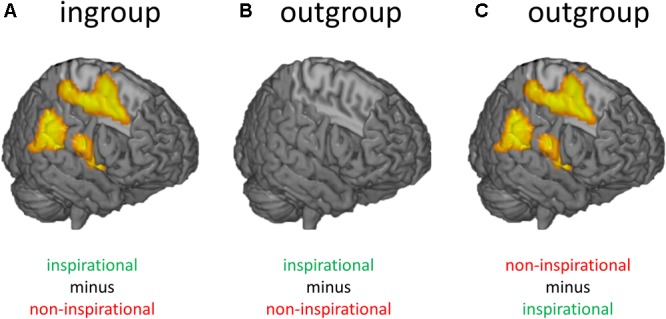
Results from an fMRI study showing how group membership influences how people process information. **(A)** Participants who believed they saw inspirational (vs. non-inspirational) statements from ingroup leaders showed more activation in brain areas involved in semantic encoding such as the rostral inferior parietal lobule, pars opercularis of the inferior frontal gyrus, and posterior midcingulate gyrus. **(B)** When the same participants believed the exact same inspirational messages came from outgroup leaders they did not show more activation in these areas. **(C)** Instead, they showed more activation in these areas for the opposite contrast (non-inspirational minus inspirational messages).

People do not only process faces in a biased way but also process the words of ingroup and outgroup members through a selective lens. Participants who identified as strong supporters of a political party rated identical statements during an fMRI experiment as more inspirational if they believed they came from ingroup (vs. outgroup) leaders (Molenberghs et al., 2017). Neuroimaging results revealed a strong interaction effect between type of statement (inspirational vs. non-inspirational) and leader (ingroup vs. outgroup leaders) in brain areas often associated with semantic processing, such as the rostral inferior parietal lobule, pars opercularis of the inferior frontal gyrus, and posterior midcingulate gyrus (Vigneau et al., 2006; Binder et al., 2009; Friederici, 2011; Torta and Cauda, 2011; Price, 2012). Two follow-up pairwise comparisons revealed that this interaction was caused by (a) increased activation for inspirational (vs. non-inspirational) statements from ingroup leaders, and (b) increased activation for non-inspirational (vs. inspirational) statements from outgroup leaders in these brain areas (**Figure 2**).

Similar biased information processing was also found in an fMRI study by Westen et al. (2006). They presented Democrats and Republicans during the U.S. Presidential election of 2004 with an initial statement (e.g., a politician said in the past that they were going to lower taxes) from a presidential candidate of their own or another political group (i.e., George Bush or John Kerry), or a politically neutral person matched for gender (e.g., an actor). Participants were subsequently shown a statement which involved an action which contradicted the initial statement (e.g., the politician is now not lowering taxes), and finally an exculpatory statement (e.g., contrary to what was previously thought, modeling now shows that there is not enough money in the budget to lower taxes and doing so would be fiscally irresponsible). Behavioral results showed that participants perceived less contradiction between the initial statement and the action that contradicted the statement from their own group leader. They were also more likely to accept an exculpatory statement if it came from the presidential candidate of their own group. Neuroimaging results revealed that contradictory statements from a political candidate from their own group (vs. the neutral candidate) led to increased activation in medial prefrontal and anterior cingulate cortex (areas often co-activated during conflict resolution; Fan et al., 2003; Domínguez et al., 2016), which was not observed in the same contrast for the political candidate from the opposing political group. Contradictory statements from the political candidate from their own group also led to more activation in left lateral inferior frontal cortex and insula (which the authors interpreted as more negative affect) compared to exculpatory statements, which was not observed for neutral targets. Together these two fMRI studies clearly show how people process information from ingroup and outgroup leaders in a biased way.

In addition to faces and words, neuroscience studies have shown that people process the actions of ingroup and outgroup members differently (e.g., Newman-Norlund et al., 2008; Gutsell and Inzlicht, 2010; Duarte et al., 2017). A famous behavioral study by Hastorf and Cantril (1954) asked opposing sport fans who was to blame for the rough play during a football game between Princeton and Dartmouth university. The game was won by Princeton and the official statistics showed that Dartmouth was penalized 70 yards and Princeton 25 yards. Princeton students saw Dartmouth players make two times as many infractions as their own team, while Dartmouth fans said both teams made the same number of infractions. The authors concluded that although both teams watched the same game, they observed a different game in their own mind. Neural evidence that people can perceive identical actions from ingroup members and outgroup members differently comes from a neuroimaging study by Molenberghs et al. (2013). Participants were randomly divided into two groups and had to compete against each other to press a button as quickly as possible. In a subsequent fMRI experiment, participants had to judge the speed of similar actions by ingroup and outgroup members. The speed of the hand actions in the videos was manipulated, so that on average, the speed of the actions of ingroup and outgroup members was the same. Behavioral results, however, revealed that, on average, participants rated the actions of own team members as faster.

The researchers separately modeled brain activations during the observation of the actions and when participants made their decision. No differences in brain activation were found when participants decided that their own (vs. other) team was faster, suggesting they were not deliberately favoring their own team. When looking at brain activation during action observation, the authors split the group into those who said that their team was, on average, faster than the other group. Neuroimaging results revealed that only participants who said that their team was faster showed more activation in the inferior parietal lobule (a critical area in the action observation network; Caspers et al., 2010; Molenberghs et al., 2012) when watching the actions of their own (vs. the other) team members. This suggests that participants who exhibited an ingroup bias in their speed ratings were perceiving the actions of ingroup and outgroup members differently. This might help explain why sport fans often gets so upset when a referee makes a decision that goes against their team (i.e., in their mind things happened differently). The results reviewed in this section show how group membership influences the neural mechanisms involved in face, word and action perception. In the next section, we will focus on how humans’ ability to empathize with the suffering of others, and the brain areas associated with this ability, are influenced by group membership.

## Reduced Empathy for Outgroup Suffering

Empathy refers to the ability to share and understand the subjective states and feelings of others. Several types of empathy are typically distinguished within the literature such as affective empathy (i.e., the ability to feel and share the emotions of others), cognitive empathy (i.e., the ability to rationally understand the emotions of others), and emotional regulation (i.e., the ability to regulate one’s emotions), with separate brain circuits associated with each type of empathy (Bernhardt and Singer, 2012; Decety, 2015). Here we focus on one particular type of affective empathy: empathy for the pain of others. The dorsal anterior cingulate (dACC) and anterior insula (AI) are consistently detected across studies in response to this type of affective empathy and respond to both the first-hand experience of pain and its perception in others (Lamm et al., 2011). One of the first fMRI studies that investigated how the neural regions involved in empathy are influenced by group membership is a study by Xu et al. (2009). They presented Chinese and Caucasian participants with video clips of Chinese and Caucasian people receiving either painful (i.e., needle prick) or non-painful (i.e., cotton swab) stimulation to the face. Observing painful stimulation of ingroup faces led to more activation in the dACC and AI, but when participants viewed outgroup faces in pain, no increased activation was observed in the dACC.

Another fMRI study that used similar stimuli examined whether a general social group category, other than race, could similarly modulate neural empathic responses and perhaps account for the apparent racial bias reported in previous studies (Contreras-Huerta et al., 2013). Using a minimal group paradigm, the authors assigned participants to one of two mixed-race teams (Chinese or Caucasian), and then measured neural empathic responses as participants observed members of their own group or other group, and members of their own race or other race, receiving either painful or non-painful stimuli. Participants showed clear group biases, with no significant effect of race, on behavioral measures of implicit and explicit group identification. Hemodynamic responses to perceived pain in dACC and AI showed significantly greater activation when observing pain in own-race compared with other-race individuals, with no significant effect of minimal groups. These results suggest that racial bias in empathic responses is not easily influenced by minimal forms of group categorization, despite the fact that participants indicated a clear increased association with ingroup versus outgroup members, as measured both by implicit and explicit measures of group identification.

Another fMRI study examined empathetic responses in soccer fans (Hein et al., 2010). Participants in this study received high or low painful shocks to the hand, and observed ingroup (i.e., fans from the same soccer team) and outgroup (i.e., fans from the opposing soccer team) members receive the same type of shocks. Activation in the AI was stronger for ingroup members in the high minus low painful condition compared to the same contrast in outgroup members, thus reflecting an ingroup bias in empathy responses. In a second session, they measured how much the participant was willing to help the ingroup and outgroup member by asking them if they were willing to receive half of the persons’ painful stimulation (and thus reduce the pain for the other person). Increased response in the AI in high versus low painful trials pooled across ingroup and outgroup conditions was associated with increased helping overall. Moreover, individual differences in the AI response in high versus low painful trials in the ingroup compared to outgroup conditions predicted how much more they were willing to help an ingroup versus outgroup member reduce their pain.

Reduced activation in the AI between watching ingroup and outgroup members in pain was also reported in an fMRI study in which White and Black participants watched video clips of white and black hands receive either painful stimulation by a syringe or non-painful stimulation by a Q-tip (Azevedo et al., 2013). Participants also completed a racial implicit association test (IAT) to measure their implicit racial bias. Watching painful stimulation to a hand from the same race resulted in increased activation in AI compared to the other race. Participants that showed a greater ingroup bias in AI activation, also showed a larger ingroup bias as measured by the IAT. Finally, a recent fMRI study suggests that perceived threat of the outgroup to the status of the ingroup can modulate ingroup bias (Richins et al., 2018). Students who watched other students from the same and other university in pain only showed less activation in AI and dACC for outgroup members if the student was from a competing university, and not if the student was from a university that was not considered a threat to the status of the ingroup.

The fMRI studies reviewed in this section suggest that people typically activate brain regions associated with watching others in pain, such as the dACC and AI, less when observing outgroup (vs. ingroup) members in pain. Individual ingroup bias differences in neural responses in these regions is also associated with reduced helping behavior (Hein et al., 2010), and increased implicit negative bias toward outgroup members (Azevedo et al., 2013). However, as the study by Richins et al. (2018) suggests, this reduced neural response when confronted with outgroup members in pain depends on the type of outgroup people are dealing with.

## Reduced Mentalizing for Outgroup Mindsets

Mentalizing (aka Theory of Mind) refers to our ability to explain and predict other people’s behavior by attributing to them independent mental states, such as beliefs, intentions and desires (Gallagher and Frith, 2003). Mentalizing differs from cognitive empathy because Theory of Mind tasks often just involve understanding another person’s mindset (e.g., false belief), without having to understand what the person is feeling (which is a fundamental component of cognitive empathy). Mentalizing relies on a network of interconnected areas in the prefrontal, temporal and parietal cortices (for recent fMRI meta-analyses see Schurz et al., 2014 or Molenberghs et al., 2016b). The two most common brain areas reliably involved in mentalizing are the medial prefrontal cortex (mPFC) and temporoparietal junction (TPJ; Van Overwalle, 2009). Neuroimaging studies suggest, however, that these two areas are not activated to the same degree in ingroup and outgroup members.

For example, Adams et al. (2010) found increased activation in the mentalizing network when thinking about the mindset of ingroup versus outgroup members during a Theory of Mind fMRI experiment. They presented Japanese and white American participants with a cross-cultural version of the Reading the Mind in the Eyes Task (RMET; Baron-Cohen et al., 2001). During the RMET, participants were presented with a picture of the eye region of a face and had to guess what the person in the picture was thinking or feeling. Half of the RMET pictures in the Adams et al. (2010) version were from Asian people and half from Caucasian people. They found that that Japanese participants performed better on the Asian version of the RMET, while the white Americans scored better on the Caucasian version. The fMRI results revealed that, when participants decoded the mindsets of people from the same (vs. other) culture, there was more activation in the bilateral posterior temporal sulcus (which was located in the TPJ area).

In another fMRI experiment, African-American and Caucasian-American participants watched pictures of African-American or Caucasian-American people in painful (e.g., in the midst of a natural disaster) and non-painful (e.g., enjoying a picnic) situations (Mathur et al., 2010). African-Americans were more willing to help ingroup than outgroup members experiencing painful situations, but Caucasian-Americans showed no difference between the two groups. Watching people in painful situations led to more activity in regions associated with empathy and mentalizing such as the dACC, AI, and mPFC. African-American participants revealed more activation in the mPFC in response to ingroup relative to outgroup unpleasant scenes compared to Caucasian-American participants. Because the African-Americans in the study identified more with their group than the Caucasian-Americans, the authors concluded that increased ingroup identification was the most plausible explanation why the African-American group showed an increased ingroup bias in behavioral ratings and mPFC activation relative to Caucasian-Americans. Another example of how culture differently influences the neural mechanisms involved in thinking about the mindsets of ingroup and outgroup members was found in a study by Cheon et al. (2011). They used similar painful and non-painful stimuli to the Mathur et al. (2010) study above but adapted the stimuli to their participants who were either South Koreans or Caucasian-Americans. South Koreans have an equally high socioeconomic status as Caucasian-Americans but they have a higher preference for hierarchical versus egalitarian social norms, compared to Americans, and a stronger preference for helping ingroup (vs. outgroup) members. This difference in social hierarchy and ingroup preference was also found in this study, with South Koreans showing a higher score on the Social Dominance Orientation (SDO) scale (Pratto et al., 1994), than the Caucasian-American participants. The behavioral results showed that South Koreans had more empathy for the suffering of ingroup than outgroup members, while Caucasian Americans again showed no such ingroup bias. Across participants, greater neural activity was found in brain areas associated with mentalizing, such as the mPFC and bilateral TPJ, when watching ingroup vs. outgroup members in pain. Interaction analyses between the two cultures showed that South Koreans showed more activation in the bilateral TPJ in response to ingroup versus outgroup members in pain compared to Caucasian Americans. Moreover, Caucasian Americans compared to South Koreans only exhibited increased activation in the lingual gyrus (but not in any of the brain areas associated with mentalizing) in response to ingroup versus outgroup members in pain. Further region of interest analyses showed that the bilateral TPJ was more active while watching ingroup (vs. outgroup) members in pain in the South Korean participants, while the Caucasian participants did not show a similar ingroup bias in this region. The activation in the left TPJ also correlated positively with the empathy ingroup bias and SDO score. The authors suggest that the difference in TPJ activity between South Koreans and Caucasian Americans might be related to their differential preference for social hierarchy and greater tendency for cooperation and altruism toward ingroup members.

Another fMRI study by Bruneau et al. (2012) presented Israelis, Arabs and South Americas participants with stories about ingroup and outgroup members in physical and emotional pain. Israelis and Arabs were considered conflict groups, and South Americans a distant group. Reading stories about physical pain activated brain areas often associated with affective empathy (i.e., AI and dACC), while reading stories about emotional pain activated brain areas often associated with mentalizing (i.e., mPFC and TPJ). No differences in brain areas implicated in affective empathy or mentalizing were found between conflict groups while reading emotional or physical pain stories, and the same was true for physical pain stories between ingroup and distant groups. However, when reading stories about ingroup members versus distant outgroup members that were suffering emotional pain, ingroup targets elicited increased activation in brain areas often associated with mentalizing such as the mPFC and right TPJ. The authors suggested that the mindset of a distant group member may be less relevant than that of a conflict group member, and that this may have been the reason why the ingroup bias was only observed in relationship to distant groups and not conflict groups.

The results in this section suggest that brain areas associated with mentalizing are typically less activated when thinking about the mindset of outgroup members (Adams et al., 2010). However, the above studies also suggest this is not always the case. For example, in the Mathur et al. (2010); Cheon et al. (2011), and Bruneau et al. (2012) studies, this reduced activation in brain areas associated with mentalizing in response to outgroup targets was only present in some groups, and these effects were associated with cultural differences. Overall, the results suggest that lower group identification (Mathur et al., 2010), lower social dominance (Cheon et al., 2011), and increased relevance of outgroup members (Bruneau et al., 2012), may lead to increased activation in brain areas associated with mentalizing in response to outgroup members.

## Increased Sensitivity for Outgroup Threats

People are usually highly sensitive to menaces and attacks from outgroup members against the ingroup because they could pose an existential threat. Attacks against ingroup members increase thoughts about one’s own mortality and lead to increased prejudice toward outgroup members (Das et al., 2009). Increased identification with a group that is a victim of a terrorist attack by an outgroup is also associated with increased fear toward that outgroup, increased behaviors to help the victims, and an increased willingness to retaliate against the outgroup perpetrators (Dumont et al., 2003). To investigate the differential effects of ingroup and outgroup attacks, Molenberghs et al. (2016a) presented participants undergoing fMRI with pictures of either an ingroup (students from the same university) or outgroup (students from a different university) member, respectively, attacking either an ingroup or an outgroup member (i.e., four different attack conditions in total). Participants rated their feelings of moral sensitivity after each interaction. The moral sensitivity score was a combination of questions asking, for instance, how much they wanted to punish the perpetrator. Moral sensitivity scores were higher in the condition where an outgroup member attacked an ingroup member, compared to the three other conditions. Participants in this condition also showed increased activation in the lateral orbitofrontal cortex (lOFC) compared to the condition where an outgroup member attacked another outgroup member. The lOFC activity associated with the outgroup attacking an ingroup member compared to an outgroup attacking an outgroup member also correlated positively with the difference in moral sensitivity score for these two conditions. These results suggest that people are highly sensitive to attacks from outgroup members against their group and that these feelings are associated with increased activation in lOFC.

Such increased activation in lOFC in response to outgroup threats was also found in an fMRI study by Dominguez et al. (2017). Here, non-Muslims were confronted with a picture of an ingroup (Western Caucasian) or outgroup (Middle Eastern Muslim) member holding either a gun or a different object. Muslims were presented stereotypically with characteristic Islamic headgear (taqiyah or kufi), beard and names (e.g., Abdul) to facilitate outgroup identification. Participants were instructed to shoot when the target was holding a gun and not shoot when the target was not holding a gun. The picture of the target was presented very briefly (250 ms) and subsequently masked so it was difficult to see which object the target was holding. Participants that had a stronger bias against Muslims, as measured by an explicit ‘attitudes toward Muslims’ questionnaire (Griffiths and Pedersen, 2009), were more likely to shoot Muslim targets and felt less guilty after shooting them. After making the decision to shoot or not shoot the target, participants were provided with feedback about the accuracy of their decision together with a picture of the face and name of the target. When people correctly shot the target, neural responses during this feedback phase showed that being confronted with an armed Muslim versus non-Muslim was associated with increased lOFC activation, thus again showing that outgroup threats are associated with more activation in lOFC. Since attacks by the outgroup may pose an existential threat, these results are compatible with studies pointing at the role of the lOFC in learning which behaviors lead to punishment and feelings of displeasure (Kringelbach and Rolls, 2004; Berridge and Kringelbach, 2013).

## Schadenfreude and Rewarding Others

Schadenfreude is a feeling of pleasure derived from another person’s misfortune. Under some circumstances (such as during war, when people are extremely disliked or when there is strong competition between the groups), harming outgroup members, or seeing them in pain or experiencing misfortune can lead to schadenfreude (Cikara, 2015). For example, when Osama Bin Laden was killed, his assassination was welcomed by many people in the West. The two most common brain areas involved in the reward system are the striatum and medial orbitofrontal cortex (mOFC; O’Doherty, 2004). Neuroimaging research has shown that when there is a strong competition between two groups, seeing outgroup members experience pain or misfortune can lead to activation in these two brain areas (Cikara, 2015).

For example, Cikara et al. (2011a) presented avid baseball fans with baseball plays of their favorite team, a rival team and neutral teams having either a successful (e.g., hitting a home run) or an unsuccessful (e.g., runner tagged out at first base) outcome. Behavioral results showed that participants experienced more pleasure when their favorite team had a successful outcome against the rival team, but also when the rival team had an unsuccessful outcome against the neutral team (the schadenfreude condition), compared to the conditions where the favorite team lost or when the neutral teams played against each other. Favorite team’s success and rival team’s failure increased activation in the striatum compared to the control condition in which neutral teams played against each other. Participants’ self-reported pleasure scores in these conditions also correlated positively with striatum activity. Reported likelihood to harm a fan of the rival team compared to a fan from the neutral team also correlated positively with striatum activity in the conditions where the rival team failed, which was in line with the authors’ hypothesis that feelings of schadenfreude were related with a desire to harm the rival group. Hein et al. (2010) found similar results in their fMRI study with rival soccer fans (which has been described in detail above in section three). They found that the stronger the striatum activation was when observing an outgroup member in pain during the first session, the less likely were they willing to help this person in a subsequent session.

Intergroup bias in the reward system is not only observed when watching others in pain but also when rewarding others. In an fMRI study (Molenberghs et al., 2014), university students had to give monetary rewards to ingroup (students from the same university) or outgroup (students from another university) members if they answered a question correctly during a trivia task. Giving rewards to both ingroup and outgroup members was associated with increased activation in brain areas involved in the reward system, such as the striatum and medial orbitofrontal cortex (mOFC), but critically, giving rewards to ingroup (vs. outgroup) members was associated with more activity in these two regions.

In another fMRI experiment (Telzer et al., 2015), Chinese and American participants could choose if they wanted to donate some of their money to ingroup or to outgroup members. Behavioral results showed that Chinese (but not American) participants donated money to the ingroup (vs. outgroup) in significantly more trials. Neuroimaging results revealed that the striatum was more active in both groups when participants donated to the ingroup versus the outgroup. Similarly, Hackel et al. (2017) found that New York university students that were highly invested in their own group (i.e, those that said that their identity as a New York university student was an important aspect of their identity) tended to give more money to people from their own group and showed more activity in the striatum when observing ingroup (vs. outgroup) gains. Finally, Bortolini et al. (2017) found that soccer fans put in more effort to gain money for ingroup members and that reward for ingroup (vs. outgroup) members was associated with increased functional connectivity between the mOFC and the subgenual cingulate cortex (SCC). The results in this section show that increased activity in the reward system can occur: 1) when observing the suffering of a competing outgroup member or 2) when giving rewards to ingroup members. These modulations of the reward system might subserve intergroup biases in several ways, from subtle forms of ingroup bias such as in a minimal group paradigm where people prefer to give more rewards to ingroup members (Tajfel et al., 1971), all the way to extreme intergroup violence where people derive pleasure from the killing of others (Grossman, 1995).

## Conclusion, Future Directions and Limitations

First, we discussed how fMRI studies have shown that our brain responds differently to faces, words and actions of ingroup and outgroup members. Depending on the context, this selective processing of ingroup and outgroup faces might be harmless or lead to subtle forms of ingroup bias. However, under some circumstances this biased processing can lead to life-or-death situations. For example, if a police officer has to make a quick decision to shoot or not shoot a target based on whether he or she believes the person is holding a gun, then it is a major problem if that decision is influenced by the target’s skin color. Behavioral results have shown that police officers in America more likely choose the “shoot” versus “not shoot” response when the target is Black compared to Latino, White or Asian target (Sadler et al., 2012). The fMRI studies on face perception reviewed above suggest that increased responses in the amygdala in response to faces from people from a different race might play a role in these quick decisions. For example, Senholzi et al. (2015) found in their fMRI study that the more non-Black American participants associated Blacks (vs. Whites) with violence and danger, the more activation they showed in the left amygdala when shooting Black (vs. White) armed targets. Future neuroimaging studies should further investigate if individual differences in “shoot” or “not shoot” decisions in front of ingroup versus outgroup members are associated with the amount of amygdala activation in response to ingroup and outgroup faces.

In relation to statements from political leaders, behavioral studies on motivated processing of political information have shown that biased processing of information can lead to a strong polarization in political attitudes (Taber et al., 2009). The two fMRI studies (Westen et al., 2006; Molenberghs et al., 2017) reviewed above suggest that neuroscience can provide some insights into clarifying how information from opposing candidates is processed. Future fMRI studies could apply these insights to better predict how messages from ingroup and outgroup leaders will be perceived (Berkman and Falk, 2013), and potentially tailor the messages to different audiences based on these predictions. Finally, the fMRI study by Molenberghs et al. (2012) on action perception suggests that people sometimes perceive the actions of ingroup and outgroup members differently, and that these perceptions unconsciously influence people’s decisions in a bottom-up manner. This might explain why sport fans get so upset about decisions against their team or why a tennis player sees their ball in and an opposing player sees the opposite. Future neuroimaging studies could explore these biases further by looking at how the strength of group identification in sport fans influences these types of biases (e.g., Do sport fans who identify more with their own team show larger perceptual biases?), or investigate when these biases turn into violent behavior (e.g., Are larger perceptual biases associated with more violent behavior toward outgroup members?).

Second, we discussed how reduced responses in the dACC and AI when seeing outgroup members in pain were associated with increased ingroup bias (Azevedo et al., 2013) and reduced prosocial behavior toward them (Hein et al., 2010). The relationship between empathy and prosocial behavior is complex. However, increased empathy for ingroup vs. outgroup members may lead people to give more resources to members from their own group (Decety and Cowell, 2015). Groups are important to humans and increased empathy for people from the same group might just be the result of an evolutionary adaptation to group living (Caporael, 1997). Research has also shown that social support from ingroup members is particularly important for people’s wellbeing (e.g., Haslam et al., 2012). Therefore, increased empathy for ingroup members in pain and increased prosocial behavior to relieve ingroup members’ suffering may be a functional response developed throughout our human history. While most fMRI studies reviewed in section three showed a reduced neural response in brain areas associated with empathy when watching people from a different group in pain (Xu et al., 2009; Hein et al., 2010; Azevedo et al., 2013; Contreras-Huerta et al., 2013), Richins et al. (2018) also showed that this reduced response depends on the relationship with the outgroup. Participants showed no ingroup bias in neural responses toward a group that was not a direct threat to the status of the ingroup. This flexibility in empathic responding to ingroup and outgroup members in different contexts is important. Despite the presence of ingroup biases, most people have the ability to empathize both with ingroup and outgroup members, even if they belong to a different ethnicity or country. However, when a conflict breaks out along ethnic lines within a country or between countries, these same people are likely to feel much less empathy for the suffering of the same outgroup members. Future fMRI studies should further investigate how these different contexts influence the neural activations associated with empathizing with others.

Third, we discussed how reduced mentalizing about the mindset of outgroup members was associated with reduced activity in the mPFC and the TPJ (e.g., Adams et al., 2010; Cheon et al., 2011). However, Bruneau et al. (2012) also showed that this ingroup bias was only found in response to distant outgroup members but not outgroup members that were in conflict with the ingroup. Another more recent fMRI study (Welborn and Lieberman, 2015) provides an alternative explanation for why people only show this bias for distant outgroup members. They found that participants who strongly identified as Republican or Democrat showed more activation in the mPFC during a trait judgment task in response to politicians they had more (vs. less) knowledge about, regardless of whether the target was from their own or the opposing political group. This suggests that increased knowledge about the outgroup member, rather than conflict with the outgroup member, might be a reason for increased activation in brain areas associated with mentalizing. Future fMRI studies should further investigate the different contexts in which people think more or less about the mindset of outgroup members and how this is associated with activation in brain areas associated with mentalizing such as the mPFC and the TPJ. In some circumstances it might be very useful to understand the mindset of an outgroup member. For example, when trying to understand the next move of an outgroup member that is trying to hurt ingroup members, more mentalizing rather than less would be functional. However, when ingroup members harm an outgroup member themselves, reduced mentalizing might be more functional.

Fourth, we reviewed that there is increased moral sensitivity for outgroup attacks on ingroup members (associated with increased lOFC activation). This suggests that there is something specific about outgroup threats toward ingroup members that can lead to strong antisocial behavior toward this outgroup. Indeed, behavioral research has shown that Islamic terrorist threats and perceived support for terrorism by Muslims are important predictors of outgroup discrimination and support for anti-immigration policies in European countries, over and above standard predictors such as prejudice and political conservatism (Doosje et al., 2009). Future fMRI studies should further investigate if different types of outgroup threats (e.g., realistic vs. symbolic threats; Stephan and Stephan, 2000) lead to similar activation in the lOFC, and if people always respond more strongly to outgroup threats regardless of the situation.

Finally, we reviewed fMRI studies showing increased activity in the striatum and mOFC when observing outgroup harm (i.e., schadenfreude) or when rewarding ingroup (vs. outgroup) members. The former usually only happens when there is a strong competition between the two groups or when the outgroup is strongly disliked. However, preferring to reward ingroup vs. outgroup members seems to happen already in minimal groups (Tajfel et al., 1971). These observations are in line with the view that ingroup bias is more about favoring the ingroup rather than harming the outgroup (Brewer, 1999; Molenberghs et al., 2014). Indeed, in most everyday situations people value their own team more and prefer that their team wins, but do not necessarily want the other team to get hurt. Future fMRI studies could research the conditions under which people like to see outgroup members being hurt, and if people always show more activation in the reward system when rewarding ingroup members. For example, activists often set up charities to support outgroup members (e.g., Westerners supporting poor children in Africa) because they feel a social responsibility for these groups and are driven by social justice (Borshuk, 2004). Are the processes that drive prosocial behavior in these situations subserved by similar neural mechanisms, and could they become more active when rewarding outgroup vs. ingroup members?

How do all of these findings fit together? The reviewed studies show that there is not a single brain area or system responsible for ingroup biases. Depending on the bias (e.g., perceptual vs. empathic bias) and the modalities (e.g., faces vs. words) implicated, different neural networks might be involved. We predict that combining multiple types of biases will lead to stronger antisocial behavior against the outgroup. For example, a perceptual bias in relation to action observation (e.g., an offensive foul during a sports game) by an ingroup member might result in seeing the action in a more favorable light than the same action performed by an outgroup member. This perceptual bias alone might not lead to violence between the two teams. However, if this perceptual ingroup bias is combined with biases in affective empathy and mentalizing, together with perceptions of threat to ingroup safety or schadenfreude for the suffering of an outgroup member, it is likely that all these biases together lead to violence between the two teams.

Why is it important to better understand the neural mechanisms of ingroup bias? First, new models extending present neuroimaging findings are important from a theoretical point of view. They provide new knowledge (e.g., Which brain areas are involved in these biases and how is neural activity modulated?), allow testing new predictions (e.g., Do people always empathize more with ingroup members and do neural responses correspond with behavioral responses?), and allow testing competing novel hypotheses that might sometimes not be possible to answer with behavioral methods alone (e.g., Are biases caused by top-down or bottom-up information processing?). Second, future studies could use these insights to target brain areas involved in intergroup bias with non-invasive brain stimulation techniques such as transcranial direct current stimulation (tDCS) and transcranial magnetic stimulation (TMS; Gallate et al., 2011; Wong et al., 2012; Gamond et al., 2017) or real-time fMRI neurofeedback (Sulzer et al., 2013) to reduce or modulate intergroup bias. Third, current neuroscience methods such as fMRI allow testing if a person is telling the truth or not (Farah et al., 2014) and predicting real-world outcomes (Berkman and Falk, 2013) with a degree of accuracy above chance level. In the future, these methods might be used to test with a high level of accuracy if a person is telling the truth about their ingroup bias or if neural responses related to ingroup bias can predict intergroup behavior in the real world beyond behavioral responses.

There are also several important limitations to the neuroimaging study of ingroup bias at present. One question to ask is whether cognitive processes can be inferred from neuroimaging data at all (for a detailed discussion on this so-called reverse inference problem see Poldrack, 2006 and Hutzler, 2014). Given that mental states generally rely on the combined activity of different brain areas and the same brain area can correlate with different mental states, activity in a given brain area does not directly provide information on a given mental state. This is especially true for high-level social processes such as intergroup bias, where there is no one-to-one relationship between a certain behavior and a particular brain area. Second, the spatial resolution of fMRI is still very low (e.g., the Blood Oxygenation Level Dependent (BOLD) signal measured in one voxel is an indirect measure of activity in hundreds of thousands of neurons), thus limiting the insights the technique can provide into the neural mechanisms involved in ingroup bias. Third, a question arises about the moral and ethical implications of using neuroimaging methods to change or predict mental processes or actions in relation to intergroup behavior. Neuroimaging techniques such as fMRI cannot reveal with 100% accuracy if a person is lying or not and cannot predict or change anyone’s behavior with a reliability level that can currently justify its use in important real-life situations (Farah et al., 2014). If reliable methods emerge, it will be important to establish moral and legal frameworks to define when it is appropriate to reveal someone’s brain processes, or to intervene to reduce their ingroup bias against particular groups. Fourth, the lack of a “neutral” control group in many of these fMRI studies makes it difficult to know if the observed bias is toward the ingroup or the outgroup. Neuroimaging studies are often limited by the number of conditions that can be presented during one single experiment and, therefore, extra control conditions are often the first to be excluded. Also, when studying groups, choosing a control group is not always straightforward. That said, some fMRI studies have included a control condition by using either an outgroup (e.g., Cikara et al., 2011b) or outgroup member (e.g., Westen et al., 2006) that was not in direct competition with the group of the participant.

While the focus of this review has been on fMRI studies, there is also a wealth of interesting insights on ingroup biases from EEG studies (see Ito and Bartholow, 2009 or Han, 2018 for reviews). Similar interesting results on ingroup biases have also been found from studying hormones such as oxytocin or testosterone (as reviewed in McCall and Singer, 2012; Cikara and Van Bavel, 2014). However, these findings are beyond the scope of this review. We also want to stress, once again, the importance of integrating multidisciplinary information from different fields to better understand intergroup bias (Decety et al., 2017).

The different ways group membership influences neural activity in the brain areas reviewed above is also not meant to be an exhaustive list of how neural modulation can lead to intergroup bias. Depending on the stimulus modality (e.g., face vs. action) and the specific cognitive process (e.g., empathy vs. mentalizing) implicated, different molecular, neuroendocrine and neural processes may be involved in creating intergroup bias (Molenberghs, 2013; Decety et al., 2017).

To conclude, the goal of this review was to bring together a wide variety of findings from fMRI studies to help explain and guide future research into the neuroscience of intergroup biases. Given the enormous consequences of positive and negative intergroup contact on a human and economic level, it is of crucial importance to acquire a better understanding of the behavioral and neural mechanisms that drive intergroup bias and violence.

## Author Contributions

PM wrote the initial draft of the paper. WL provided substantial contributions to the final version of the paper.

## Conflict of Interest Statement

The authors declare that the research was conducted in the absence of any commercial or financial relationships that could be construed as a potential conflict of interest. The reviewer CP and handling Editor declared their shared affiliation at the time of the review.
